# Carrageenan biosynthesis in red algae: A review

**DOI:** 10.1016/j.tcsw.2023.100097

**Published:** 2023-01-21

**Authors:** Antonin Chevenier, Diane Jouanneau, Elizabeth Ficko-Blean

**Affiliations:** Sorbonne Université, CNRS, Laboratory of Integrative Biology of Marine Models (LBI2M), Station Biologique de Roscoff (SBR), 29688 Roscoff, Bretagne, France

**Keywords:** Red algae, Carrageenan, Sulfotransferases, Glycoside hydrolases, Glycosyl transferases, Biosynthesis, Cell wall

## Abstract

In this review, we summarize the current state of knowledge on the biosynthesis of carrageenan by exploring both the enzyme activities and their localizations. Genomic data, with the sequencing of the genome of *Chondrus crispus* and the first transcriptomic study into the life cycle stages of this organism, as well as fine carbohydrate structural determination of matrix glycans, provide leads in the study of carrageenan anabolism. Comparison to related carbohydrate-active enzymes, detailed phylogenies alongside classic histochemical studies and radioactivity assays, help predict the localization of the carrageenan-related enzyme biochemistries. Using these insights, we provide an updated model of carrageenan biosynthesis which contributes to understanding the ancestral pathway of sulfated polysaccharide biosynthesis in eukaryotes.

## Introduction

Red algae are ancient photosynthetic eukaryotes with both unicellular and multicellular species. They are the earliest known extant example of complex multicellularity (Bengtson et al., 2017) and hold an early diverging position in the Archaeplastida. Red macroalgae possess an extracellular matrix (ECM) consisting of a complex supramolecular network connecting the cells that bestows structural integrity, and functions in communication, development and defense (Kloareg et al., 2021). The ECM structures in red algae vary depending on species and life cycle stage; however, a main component is often the complex sulfated galactans such as agars, porphyrans and carrageenans (Ficko-Blean et al., 2015). Due to their particular polyanionic composition, sulfated polysaccharides retain water and are sometimes referred to as phycocolloids, with gelling and viscosity properties that vary depending on the structural modifications on the polymer. These physical characteristics play fundamental roles in the adaptation of algae in the marine environment, protecting against desiccation and maintaining flexibility in strong ocean currents and waves (Kloareg and Quatrano, 1988).

Carrageenans are sulfated galactans that constitute one of the most abundant constituents in the ECM of carrageenophyte red algae. These polysaccharides are linear and consist of the assembly of a repetitive disaccharide pattern (carrabiose) composed of β-d-galactose (G-unit) (1,4)-linked with an α-d-galactose (d-unit) or a 3,6-anhydro-α-d-galactose (DA-unit). The carrabiose units are linked by an α-1,3 glycosidic bond and several motifs exist due to the variability in the number and the position of the sulfate esters and by the presence or absence of the 3,6-anhydro-bridge on the α-d-galactose moiety (Knutsen et al., 1994). It is the presence of the 3,6-anhydro-bridge which drives gel formation in carrageenans. The mature carrabiose motifs founds in the ECMs of carrageenophyte red algae are β- (G-DA), κ- (G4S-DA), ι- (G4S-DA2S) and λ- (G2S-D2S,6S) carrabiose.

The particular chemical structure and great variability of carrageenans give them properties that have been exploited in industrial applications. In the food industry, carrageenan polymers constitute one of the major texturizing agents and are used as thickening, gelling, emulsifying or stabilizing agents in dairy, dessert, beverage and meat preparations (McHugh, 2003, Necas and Bartosikova, 2013, Piculell, 2006). They are used in various non-food products like shampoos, toothpastes, cosmetic creams and even firefighting foam (Necas and Bartosikova, 2013). Other uses for carrageenans have been found in biomedical applications. Indeed, due to their very low toxicity and rheological properties, many biocompatible composites polymers with carrageenan have been developed for drug delivery systems and tissue engineering (Genicot et al., 2018, Zia et al., 2017). Carrageenan polymers and their derivative oligosaccharides have also been shown to exhibit many biological activities of interest like antihyperlipidemic, anticoagulant, antiviral, antitumoral and anti-inflammatory activities (Genicot et al., 2018, Necas and Bartosikova, 2013, Sokolova et al., 2014) though intestinal inflammation may be induced when ingested (Tobacman, 2001). Interestingly, carrageenans can also act as biostimulants in agriculture (Lemonnier-Le Penhuizic et al., 2001).

## A putative biosynthetic pathway

A carrageenan biochemical synthesis pathway was proposed in 1979 (Craigie and Wong, 1979) and updated in 2015 (Ficko-Blean et al., 2015). Most steps are hypothetical and are based on the knowledge of the chemical diversity of carrabiose units. The biosynthetic scheme predicts three main types of enzymatic activities: galactosyltransferase (GT), carbohydrate-sulfotransferase (CST), and galactose-sulfurylase (GS). Glycoside hydrolase (GH) activity may also be involved in ECM modification, such as during cell division. Biochemical studies on recombinant red algal biosynthetic enzymes such as GSs, GTs and CSTs are challenging, mainly due to protein production problems (this is painful personal knowledge). The only biochemically characterized carrageenan-related red algal enzymes have been obtained from extracts of *Chondrus crispus* (Genicot-Joncour et al., 2009, Wong and Craigie, 1978, Zinoun et al., 1997) and *Solieria chordalis* (Goulard et al., 2003). Recently a recombinantly produced β-porphyranase was biochemically characterized in *C. crispus*, though it is uncertain whether this enzyme is related to algal defense or specific for specialized motifs found within its own ECM (Manat et al., 2022).

**Fig. 1:**
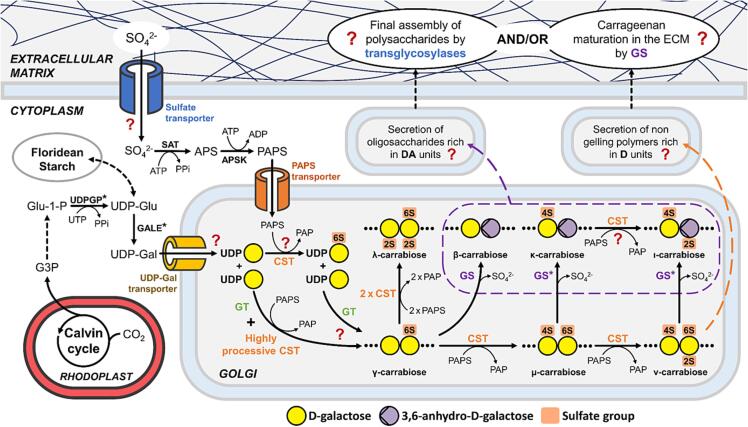
Schematic representation of the putative biochemical pathway and localization of carrageenan biosynthesis. Question marks indicate open questions into the biosynthetic step. It remains unsure if formation of the 3,6-anhydro-bridge is catalyzed in the Golgi apparatus or in the extracellular matrix or both, thus the reactions within the dashed purple are not necessarily found in the Golgi apparatus. An asterisk (*) is used to show where biochemical activities have been demonstrated in red algae. APS: adenosine-5′-phosphosulfate; APSK: APS kinase; CST: Carbohydrate-sulfotransferase; G3P: Glyceraldehyde-3-phosphate; GALE: UDP-glucose-4-epimerase; GS: Galactose-sulfurylase; GT: Galactosyl-transferase; PAPS: 3′-phosphoadenosine-5′-phosphosulfate; PAP: 3′-phosphoadenosine-5′-phosphate; PPi: Pyrophosphate; SAT: Sulfate adenylyl-transferase; UDP-Gal: Uridine diphosphate galactose; UDP-Glu: Uridine diphosphate glucose; UDPGP: UDP–glucose pyrophosphorylase.

The formation of the β-(1,4) and α-(1,3) glycosidic bonds during polymerization of galactose into the carrageenan backbone is presumably catalyzed by GTs using UDP-galactose obtained from epimerization of UDP-glucose by UDP-glucose-4-epimerase (Fig. 1). This enzyme has been characterized biochemically in the red algal carrageenophyte *S. chordalis* (Goulard et al., 2003) as has the UDP-glucose-pyrophosphorylase activity (Goulard et al., 2001). In *C. crispus,* one candidate gene for the UDP-glucose-4-epimerase has been identified (Collén et al., 2013). Two scenarios for the polymerization of the backbone have been proposed (Ficko-Blean et al., 2015). In the first case, the β-(1,4) and α-(1,3) GT steps may use the substrate UDP-galactose leading to the synthesis of a neutral galactan, followed by sulfurylation by a highly processive CST to form γ-carrageenan (G-D6S). The only paper we identified describing a neutral 4-linked α-d-galactose in carrageenan motifs was by Estevez et al (Estevez et al., 2000) which supports this first hypothesis. In the second theoretical possibility, the assembly of the polymer may result from GT reactions using UDP-galactose and UDP-galactose-6-sulfate as substrates to form the γ-carrageenan (Fig. 1). Several multigene families of GTs have been identified in *C. crispus* to be homologues to the animal GTs responsible for the biosynthesis of glycosaminoglycans (GAGs) (Breton et al., 2012). This suggests the algal enzymes are also involved in sulfated polysaccharide biosynthesis. These GTs have been discussed extensively in Lipinska et al, Ficko-Blean et al and Collén et al.

In *C. crispus*, seven genes encoding CSTs have been found and their closest non-algal homologues are metazoan CSTs involved in the regiospecific sulfurylation of GAGs (Collén et al., 2013, Ficko-Blean et al., 2015, Kloareg et al., 2021, Lipinska et al., 2020). Regarding the variety of sulfate positions in carrageenan, it is likely that most of these candidate algal CSTs also intervene in the biosynthetic pathway in a regiospecific manner. This hypothesis is supported by the developmental stage variation of *C. crispus.* In gametophytes, the main motifs are 4-sulfated with κ- (G4S-DA) and ι- (G4S-DA2S) carrabiose, whereas tetrasporophytes are λ-predominant (G2S-D2S,6S) (McCandless et al., 1973). Since G4S is found mainly in gametophytes and G2S is found in tetrasporophytes, the activities of the CSTs responsible are regulated differently between life stages. Transcriptomic analyses of differentially expressed carbohydrate-active enzymes (CAZymes) in *C. crispus* identified four CST genes that were differentially expressed between gametophytes and tetrasporophytes (Lipinska et al., 2020). These results indicate that at least some variation in carrageenan composition between life stages is regulated at the gene expression level.

Sulfate available in the marine environment is ubiquitously present in the form of inorganic sulfate (SO_4_^2-^), which must be incorporated into an activated form for its transfer onto carrageenan. In eukaryotes, the main sulfate donor is 3′-phosphoadenosine-5′-phosphosulfate (PAPS). In terrestrial plants, PAPS is generated from ATP and SO_4_^2-^ through the sequential activity of two distinct enzymes: sulfate adenylyltransferase (ATP sulfurylase), which transfers AMP from ATP to sulfate to form adenosine-5′-phosphosulfate (APS); and APS kinase, which phosphorylates APS on the 3′-OH to form PAPS (Günal et al., 2019). In animals, these enzymes are found on one polypeptide chain. Similar to terrestrial plants rather than animals, the two enzymes necessary for PAPS biosynthesis were identified in *C. crispus* (Fig. 1) (Collén et al., 2013). In the red alga *Rhodella maculata*, AP^35^S and traces of PAP^35^S were identified after incubation of the soluble lysate with ATP and ^35^SO_4_^2-^, supporting PAPS as the donor molecule for the CSTs (Møller and Evans, 1976). In metazoans, PAPS is transported from the cytosol into the Golgi via a specialized PAPS transporter, where sulfurylation during GAG biosynthesis occurs (Dick et al., 2012, Prydz, 2015). One gene encoding a putative PAPS-transporter has been identified in *C. crispus* (Collén et al., 2013) which supports sulfurylation occurring in the Golgi apparatus.

The formation of the 3,6-anhydro-bridge on the DA residue is catalyzed by the GSs. The conversion of μ- (G4S-D6S) to κ- (G4S-DA) carrageenan was observed from a *C. crispus* extract (Wong and Craigie, 1978) and led to the first biochemical hypothesis of the carrageenan biosynthetic pathway (Craigie and Wong, 1979). GS activity was detected on medium length carrageenan precursors and not smaller oligosaccharides, indicating a preference for polymers (Zinoun et al., 1997). More recently, two galactose-2,6-sulfurylases (I and II) were purified from a *C. crispus* extract and biochemically characterized (Genicot-Joncour et al., 2009). These two enzymes are classified as d-gal-2,6-sulfurylases as they have been shown to catalyze the conversion of ν- (G4S-D2S,6S) into ι- (G4S-DA2S) carrageenan but with different modes of actions. Sixteen GS-II genes have been annotated in *C. crispus*, this represents a rare example of a multigenic family in this organism and likely reflects the importance of GSs in red algal metabolism (Collén et al., 2013, Lipinska et al., 2020). Unexpectedly, transcriptomic analyses showed that ten of the GS-II genes are upregulated in *C. crispus* tetrasporophytes which are λ-carrageenan dominant (G2S-D2S,6S) and diminished in DA residues. Because natural carrageenans are hybrid polymers, it has been suggested that these GSs might be involved in the formation of punctual motifs containing the 3,6-anhydro-bridge in tetrasporophytes which may play a role in recognition, signaling or developmental events (Lipinska et al., 2020). The same transcriptomic analyses showed that only one GS-II gene was significantly upregulated in the *C. crispus* gametophytes, which are DA rich, relative to tetrasporophytes.

## Composition of the polysaccharide chain

The action of the carrageenan biosynthetic enzymes must have a direct impact on the composition of carrageenans (*i.e.* the presence of various motifs) but also on their distribution along the polymer. The hybrid nature of carrageenans was first demonstrated by comparing the rheological properties of carrageenans from selected species to mixtures of homopolymeric carrageenans (Van de Velde et al., 2005). Later, the use of carrageenan-specific marine bacterial endo-hydrolases corroborated this result and made it possible to describe the hybrid distribution of carrabiose motifs along the carrageenan chains (Guibet et al., 2008).

In depth structural analysis of κ/β-carrageenan from *Tichocarpus crinitus* and *Furcellaria lumbricalis* (Correc et al., 2012) highlighted very different motif distributions. A block distribution of κ- and β-carrabiose motifs in *T. crinitus,* and a seemingly arbitrary distribution in *F. lumbricalis*. This could be explained by two different modes of action of the CSTs acting in these two species: processive in *T. crinitus* and random (but still regiospecific) in *F. lumbricalis*. The very low amount of γ- and μ-carrabiose detected in both species suggests a processive mode of action of the GSs.

The distribution of precursor μ- and ν-carrabiose in κ-, ι- and hybrid κ/ι-carrageenans has also been studied. Distribution of μ-carrabiose was not random in *Kappaphycus alvarezii* (Jouanneau et al., 2010b), which suggests a processive mode of action of at least one of the GS. On the contrary, ν-carrabiose in *Eucheuma denticulatum* seemed randomly distributed along ι-carrageenan chains (Jouanneau et al., 2010a) suggesting a more random or at least less processive mode of GS action. Hybrid carrageenans from *Chondracanthus chamissoi, Mazzaella laminarioides, Sarcothalia crispata, and Sarcothalia radula*, containing κ-, ι-, μ- and ν-carrabiose, were also examined. After polymer treatment with marine bacterial carrageenases, many types of hybrid oligos were characterized but not κ/ν-carrabiose together nor ι/μ-carrabiose together (Jouanneau et al., 2011). The hypothesis of a common pathway for these motifs (Fig. 1) is likely as these structural results suggest that: (1) κ- and ν-carrabiose are synthesized from the same μ-carrabiose precursor, which is why they haven’t been structurally characterized adjacently and that (2) ι-carrabiose is synthesized from ν-carrabiose, as has been biochemically demonstrated via the activity of the galactose-2,6-sulfurylase (Genicot-Joncour et al., 2009), otherwise we would expect to find some μ-carrabiose adjacent to the ι-motifs. However, this doesn’t completely discount the possibility of an CST converting κ- to ι-carrabiose via an alternate pathway as well (Fig. 1).

In the structural characterization of λ-carrageenan, an unusual minor motif, bearing four sulfates per disaccharide unit (G2S,4S-D2S,6S), has been described (Guibet, 2007). The additional sulfate, modified on a lambda motif, was present on the C4 of the galactose unit, which might suggest that the same CST is produced and active in both gametophyte and tetrasporophyte in *Sarcopeltis* (*Gigartina) skottsbergii* or that there is more than one CST active with regioselectivity for the G4 position.

## Cellular localization of carrageenan biosynthesis

The specific locations of carrageenan biosynthetic enzymes remain highly hypothetical thus we will discuss them in context of carrageenan localization and biosynthesis. Histochemical and autoradiographic experiments of sulfate incorporation and localization in the red algae *Eucheuma nudum* (La Claire II and Dawes, 1976) proposed the export of a neutral galactan with subsequent sulfurylation by CSTs in the ECM since they didn’t detect ^35^S in the Golgi apparatus. However, subsequent autoradiographic experiments in *C. crispus* showed the presence of ^35^S in the Golgi vesicles (Tveter-Gallagherl et al., 1981). Pulse chase experiment showed rapid movement of ^35^S in through the endoplasmic reticulum and Golgi apparatus to the ECM and extraction of carrageenans confirmed ^35^S labelling (Tveter-Gallagher et al., 1984). In metazoans, GAG/proteoglycan biosynthesis is performed by GTs and CSTs in the Golgi where chain elongation and sulfurylation takes place (Dick et al., 2012, Prydz, 2015). Given the divergent evolution between some red algal GTs and animal GTs implicated in GAG biosynthesis as well as red algal CSTs and their CST homologues in animals (Breton et al., 2012, Collén et al., 2013, Ficko-Blean et al., 2015, Kloareg et al., 2021, Lipinska et al., 2020), it would be consistent that the mechanism of sulfated polysaccharide secretion be conserved between animals and algae. Further supporting this relationship, sulfated polysaccharide biosynthesis has been demonstrated in the Golgi of brown algal secretory cells (Callow and Evans, 1976).

Regarding the formation of the 3,6-anhydro-bridge by GSs, this maturation step may take place in Golgi vesicles or within the ECM or possibly both (Fig. 1). Immunolocalization with anti-ι-carrageenan antibodies localized to the *trans*-Golgi in *Agardhiella subulata* (Gretz et al., 1990). Monoclonal antibodies against κ-, ι-, λ-carrageenan and precursors have shown these epitopes in intracellular compartments of *Kappaphycus alvarezii* which suggested at least some intracellular activity of GSs (Vreeland et al., 1992). However, Vreeland et al describe that after alkaline treatment, which can chemically form the 3,6-anhydro-bridge in 6-sulfated-galactose, κ-antibodies labelled more strongly the intracellular carrageenans in medullary cells; whereas with no chemical treatment, the antibody against precursor carrageenans labeled more strongly (Vreeland et al., 1992). Some valid concerns have been raised concerning the full polymerization of κ- and ι-carrageenan gelling motifs intracellularly (Vreeland and Kloareg, 2000). If the GSs are active in the Golgi, the formation of polymers rich in DA residues could induce the formation of gelled material inside the intracellular vesicles. It is possible that red algal cells could synthetize smaller soluble 3,6-anhydro-containing oligosaccharides in their vesicles for extracellular incorporation into higher molecular weight polysaccharides in the ECM by as yet identified transglycosylases (Fry, 1995) (Fig. 1). Protoplasts of *K. alvarezii* were described as secreting fragments of ι-carrageenan into the medium to regenerate their ECM, though the samples were tested 24–48 h after protoplast isolation and not immediately upon release (Zablackis et al., 1993). Moreover, low molecular weight polymers containing κ-, ι-carrabiose, precursors, agar motifs, including dl-hybrids, have been characterized from *K. alvarezii* (Estevez et al., 2004, Estevez et al., 2000). However some consideration must be taken as some marine bacteria, particularly among the Bacteroidota, are known degraders of algal polysaccharides (Barbeyron et al., 2016). Alternatively, it is possible that the red algal cell might export non-gelling polymers containing mainly precursor motifs to the ECM. The polymers would thus be matured directly in the ECM by secreted GSs (Fig. 1) in order to acquire their gelling proprieties (Vreeland and Kloareg, 2000).

## Conclusions and perspectives

The particular chemistry of carrageenans as well as their heterogeneity makes them a challenge to study from the point of view of their biosynthesis. There remain many future challenges to experimentally demonstrate carrageenan-related biochemistries, cellular locations of the enzyme activities and biological functions using genetic techniques such as CRISPR-Cas9 which has recently been successful in brown algae (Badis et al., 2021). The divergent evolutionary relationship between *C. crispus* GTs and CSTs and those involved in GAG biosynthesis in metazoans (Breton et al., 2012, Collén et al., 2013, Ficko-Blean et al., 2015, Kloareg et al., 2021, Lipinska et al., 2020) also supports a common origin of sulfated polysaccharide biosynthesis in eukaryotes. To this end, resolving the mechanisms of galactan synthesis in red algae would greatly improve our understanding on the evolution of extracellular matrices in eukaryotes.

## CRediT authorship contribution statement

AC wrote the first draft, made the figure and helped with editing. DJ contributed analysis and writing to the original document. EF-B was involved in writing, editing and supervision of the project.

## Declaration of Competing Interest

The authors declare that they have no known competing financial interests or personal relationships that could have appeared to influence the work reported in this paper.
